# A Mixed Methods Study of 15 Years of Aboriginal Health Research in the Kimberley: ‘We’ve Been Researched, We Think, from Head to Toe, Inside and Outside, Upside Down’

**DOI:** 10.3390/ijerph20064823

**Published:** 2023-03-09

**Authors:** Allaneh Matsumoto, Kelly Blackburn, Billy Spicer, Kimberley Seear, Charmane Johnson

**Affiliations:** Kimberley Aboriginal Health Research Alliance, Kimberley Aboriginal Medical Services, Broome, WA 6725, Australia; allaneh.matsumoto@kahra.org.au (A.M.); spicerbilly51@gmail.com (B.S.); kimberley.seear@kahra.org.au (K.S.); charmane.johnson@kahra.org.au (C.J.)

**Keywords:** Aboriginal, Indigenous, research, health, wellbeing

## Abstract

Indigenous peoples have long been the subjects of research, with the burden generally outweighing the benefit. This mixed methods study investigates the characteristics and outcomes of Aboriginal health research in the Kimberley region of Western Australia from 2006–2020 to inform future research practices. Quantitative data from projects submitted to the Kimberley Aboriginal Health Planning Forum Research Subcommittee were reviewed, and key characteristics were recorded and descriptively analysed. Fifteen individuals from a range of local organisations who were involved with research during this time participated in qualitative semi-structured interviews, including 11 Aboriginal people. The project team, including Aboriginal investigators, integrated quantitative and qualitative findings. Three major interview themes were questionable ‘research world’ behaviours; translation of findings and impact of research; and local involvement and control. The experiences of interviewees were congruent with quantitative data for the larger body of projects (*N* = 230). Most projects (60%) were not initiated within the Kimberley, with positive impact for local communities often not clear. There were, however, examples of Kimberley Aboriginal-led research excellence. A way forward includes research that is developed, driven, and led by the community; alignment with research priorities; local Aboriginal involvement that is resourced and acknowledged; and comprehensive knowledge translation plans embedded in projects.

## 1. Introduction

Aboriginal peoples in Australia are the longest continuous surviving cultures in the world. They have never stopped fighting for their people, their cultures, and their self-determination in the face of colonisation [1,2]. There is a long history of researchers using Indigenous peoples in Australia and internationally for research gain without adequate benefit for the communities within a context of historical and contemporary colonisation [3,4,5,6]. This includes health research with Indigenous peoples, which should contribute to improved health. There is various literature detailing the problems and necessary improvements in the conduct of such research in Australia and internationally (e.g., [3,4,7,8,9,10,11]), yet problems remain. However, positive trends are gaining momentum in this space with the introduction of ethical and local guidelines for research, increased emphasis on knowledge translation, and the expectation of community involvement and leadership by Aboriginal and Torres Strait Islander people in Aboriginal and Torres Strait Islander health research [12]. 

The Kimberley region of Western Australia was one of the final areas targeted by European settlers in the invasion of Australia, and stories of the strength and determination of Kimberley Aboriginal people have a long history [1]. The Kimberley is a heavily researched region. In 2006, the Kimberley Aboriginal Health Planning Forum (KAHPF) Research Subcommittee was formed with the aim of improving research processes and encouraging appropriate research [13]. Projects need to be supported by this Subcommittee before being reviewed by the Western Australian Aboriginal Health Ethics Committee (WAAHEC). In 2013, the KAHPF Research Subcommittee completed a review of its processes, including a retrospective file audit of its records, including regional stakeholder feedback on projects that were submitted [13]. In addition, a review form was sent to researchers to gain insight into their experiences with the KAHPF Research Subcommittee and conducting Kimberley research. The findings from this review highlighted the need to prioritise research that addresses local need, for greater interconnectivity of local organisations and researchers, and suggested that changes to institutional timeframes and funding processes could improve community-based research. It was noted that local knowledge and establishment of local relationships must be built into projects for research to benefit the community, which contrasted with frequent experiences of external researchers attempting to conduct research without any regional input. 

The current study extends this previous work in the Kimberley by examining projects submitted to the KAHPF Research Subcommittee until the end of 2020. Exploring the experiences of organisations with research and what changes their representatives would like to see in this space. This is an establishment project of the Kimberley Aboriginal Health Research Alliance (KAHRA). KAHRA was formed by health services and research institutes within the region with a vision of Aboriginal people in control of Aboriginal health research to improve Aboriginal health and wellbeing in the Kimberley. The aim of this project was to investigate the characteristics and outcomes of Aboriginal health research in the Kimberley between 2006–2020 to inform future research practices in the region. The objectives were to:

- Describe the characteristics of research teams and research projects conducted in the Kimberley region.
- Identify the major themes and outcomes of the body of research and compare these to current Kimberley research priorities.
- Explore the experiences of stakeholders involved in research in the region and document recommendations regarding processes and translation.

## 2. Materials and Methods

### 2.1. Study Design

**Figure 1:**
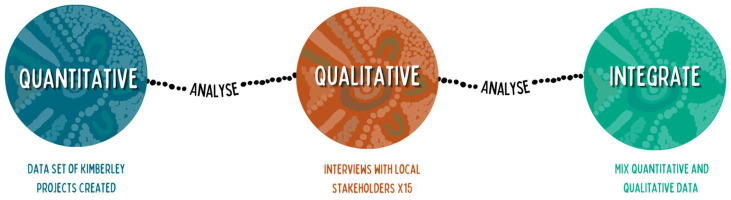
Diagram of explanatory sequential mixed methods.

An explanatory sequential mixed methods approach using both quantitative and qualitative methods (Figure 1) was used in this study [14]. This was underpinned by a pragmatic approach and an action-oriented transformative worldview [15]. The pragmatic approach allows for multiple ways of conducting processes within a research project and can be molded to the varying needs of different projects. In the transformative worldview, emphasis is placed on changing individuals’ lives and reducing the impact of issues affecting them [15]. The theoretical framework underpinning this research is postcolonial theory—how the history of colonisation has and continues to shape people’s lives, especially Aboriginal people [16]. As described by Browne et al. [16], the ‘post’ of postcolonial can be attributed to the movement of increasing Aboriginal control over research and research practices over the past few decades that still continues. At least one Aboriginal investigator was present at each interview and at every stage, from qualitative coding to the final write-up. The Aboriginal investigators were also involved in quantitative data collection and interpretation to provide cultural oversight and crucial input for the entire project [17].

### 2.2. Setting

The Kimberley region is situated in the far north of Western Australia. It spans an area of more than 400,000 square kilometres [18], containing six towns and over 150 communities, with vastly different physical and cultural landscapes. The estimated population is approximately 36,000 people, with approximately 14,000 of these identifying as Aboriginal [18]. The region has high levels of socioeconomic disadvantage, as well as poor health conditions [19]. The Kimberley has many strong and diverse cultural groups and community-controlled organisations that work together for the self-determination of Kimberley Aboriginal people [20]. 

### 2.3. Participants

Projects with applications submitted to the KAHPF Research Subcommittee from its formation in 2006 until the end of 2020 were included in the quantitative component of this project. The earliest year assigned to application reference numbers was 2007. 

For the qualitative component of the project, individuals from a range of Kimberley-based health and community organisations were invited to participate in interviews. These individuals were known by the investigators to have been involved in health research in the Kimberley in the last 15 years, including being identified through the quantitative component of this project (listed as investigators on projects, for example). The participants were in a range of work roles at various levels and also varied in their research experience and involvement. Experience ranged from being consulted about potential research projects to being an investigator on multiple varied projects and contributing to the oversight of research processes at regional, state, and national levels. Of the 15 participants, 11 were Aboriginal people; the non-Aboriginal participants had all lived and worked in the Kimberley for well over 10 years. A total of four people did not respond when invited via email for an interview. No one declined involvement. Interviews were concluded when there were indications of saturation [21], and COVID-19 impacts also meant the project team did not feel it appropriate to burden staff or the community with an additional workload. 

### 2.4. Data Collection

Quantitative data were collected from all KAHPF Research Subcommittee application forms, annual reports, and final reports available at the end of 2020 by KB with assistance from CJ. KB contacted investigators and organisations from projects with unclear completion status and requested outstanding reports (approximately 130 projects). Data items were: year of application; the number of investigators listed; the number of Kimberley-based investigators; the number of Aboriginal investigators; whether the project was initiated within the Kimberley; location/s within the Kimberley; topic; purpose; funding source; funding amount; status of the project; and year of completion if applicable. This was a time-consuming process due to limited existing accurate KAHPF Research Subcommittee summary data, while various pieces of information were unable to be sourced for many projects. This was associated with the lack of dedicated resourcing for the KAHPF Research Subcommittee secretariat role until late 2020. Interviews were conducted between November 2021 and January 2022 by a combination of authors KB, AM, and BS. KB is a non-Indigenous woman who grew up in Broome, AM is an Aboriginal woman from the West Kimberley, and BS is an Aboriginal man from the Northern Territory. At least two of these investigators conducted each interview and at least one of the Aboriginal investigators (BS and AM) co-led each interview. Interviews were conducted at the workplace of the interviewee or interviewers. Three interviews were conducted via Microsoft Teams videoconferencing for participants that did not live close to Broome. Ideally, all interviews would have been conducted face to face, but COVID-19 travel restrictions at the time did not allow for this. Participants provided informed written consent to be interviewed, and each participant consented to audio recording. 

The semi-structured interviews consisted of six main questions with prompts for each within the domains of context, process, impact, and future (Table A1 in Appendix A). While in this form of research, the quantitative phase is usually directly linked to follow-up components of the qualitative phase, in this instance the quantitative phase served as a general guide for questions directed at people in the Kimberley that had been involved in research in the Kimberley. Individual questions were not aimed specifically at certain people depending on which projects they were involved in. Interviews were conducted in an informal manner with high levels of rapport. Most interviewees were known to the interviewers through professional or personal networks. As noted by Jamieson et al. [8], familiar interviewers with established rapport are beneficial for research projects such as this. All participants were provided with a copy of their transcript and given the opportunity to make any changes. One participant made changes to give greater context to components of their transcript, and another participant made very minor changes. 

### 2.5. Data Analysis

Quantitative data were cleaned and descriptively analysed by author KS using Stata statistical software 17 (StataCorp 2021) in collaboration with author KB who had the greatest familiarity with the raw data. The analyses and preliminary findings were discussed with the other co-authors throughout this process.

Interviews were transcribed verbatim by authors KB and AM. In addition to authors AM, BS, and KB (described above), authors CJ and KS contributed to the qualitative data analysis. CJ is an Aboriginal woman from the East Kimberley, and KS is a non-Indigenous woman who has lived in the Kimberley for around 10 years. All members of the research team were living in Broome and employed by the Kimberley Aboriginal Health Research Alliance through Kimberley Aboriginal Medical Services, with strong regional understanding and relational accountability. The perspectives of the Aboriginal members of the research team were prioritised throughout the analysis process, which took place over 14 weeks. KS provided methodological oversight as an experienced researcher. Descriptive coding [22] of the interviews was conducted by the research team using NVivo qualitative data analysis software (Release 1, QSR International). Codes were then cleaned and refined, and combined where it was apparent that they could be sub-codes of a potentially emerging theme, using an inductive approach. Preliminary themes were compared to the quantitative findings and discussed within the research team. These themes were checked against the transcripts and agreed by the research team to be an accurate reflection of the interview data [23]. Relevant quantitative data were integrated within each of these themes through consensus, with clear consistency between the two data types [15]. Illustrative quotes were jointly chosen by the research team. Direct quotes were used where possible to respect and promote participant voices, prioritising Aboriginal participants. The research team considered that the quote used in the title reflected the key findings of this project. The results were drafted by KB collaboratively with the other research team members and refined together in an iterative process discussed by Dr. Michelle Kennedy [24]. A similar process was used for the discussion section after the main messages were agreed upon by the research team. All participants were given a copy of the results of this project before being released to stakeholders and the public to ensure they were comfortable with how the results were analysed and written up by the research team. This extra layer of rigor ensured that the participants were involved at each stage and that the validity of their voices was maintained through analysis and write-up. SRQR guidelines were also used to guide the qualitative reporting in this project [25].

## 3. Results

Three major themes in the interview data were questionable ‘research world’ behaviours; translation of findings and impact of research; and local involvement and control. The experiences of interviewees were congruent with quantitative data for the larger body of Kimberley research projects. Additional quotes are included in Table A2. 

### 3.1. Questionable ‘Research World’ Behaviours

Although interviewees were not directly asked about negative experiences with research and similar projects, all reported these. This included specific examples and more general dissatisfaction with the typical nature and volume of research conducted in the region. Table 1 shows the high number of projects submitted to the KAHPF Research Subcommittee, totalling 230 in 14 years. This included at least 172 completed and current projects.

“We’ve been researched, we think, from head to toe, inside and outside, upside down … I think that people just getting tired of being researched, there’s no follow up.”

(Participant 11)

“That’s the thing with research, people can tell you in the community, a lot of people will tell you what they want, what they need for nothing, you don’t pay, people will get big funding to go and do research. And are still the same … community people, a lot, a lot of the stuff people ask, and they can tell you what the issues are. But unless it’s in a research thing, or something, it seems like nobody’s taking notice of it, or someone’s going to get the big money to come and do the research to say what people are telling us.”

(Participant 15)

As shown in Table 1, projects received funding from a range of sources, including the WA and Australian Government Departments of Health and the National Health and Medical Research Council (NHMRC). Fifteen out of the 27 projects with “indeterminate” status (unable to be ascertained) were known to have received funding.

A high-profile regional advocate said they have not always felt “listened to” by researchers, and there was a disconnect between “a research world” and “the world we live in” of providing primary health care. This interviewee also discussed that simple ideas from communities are sometimes influenced by researchers, which “confuses what the community’s intent was … sometimes it’s just a simple research, doesn’t have to be this big to get an outcome” (Participant 10). Another interviewee expressed that “I feel like a lot of researchers, the more senior that they are as well, just get so, it’s just that real ego, about a lot of them. And so, you know, they chuck hissy fits when we say no to them, or we challenge them about things” (Participant 1). 

Some interviewees spoke about researcher communication lacking transparency, including during initial attempts to engage with individuals, communities, and organisations in the Kimberley. This included receiving explanations early in the project that were not followed by adequate communication as the researchers added more to the project, which involved additional burden on these interviewees’ organisations, or researchers initially stating, “we’re not really researching”. Another participant recounted that “I think we saw the benefit in it. But then the way that things sometimes got done, didn’t really align with the story that we were told at the start” (Participant 1). 

Other interviewees stated that at the time of initial engagement, most researchers already had the project planned, and the interviewees were unable to have input into the research plans from a local perspective. Several interviewees discussed that initial engagement from researchers had generally been to acquire a support letter for funding or ethics applications. Further, other interviewees stated that research ideas tend to come from a personal curiosity of researchers, without thinking about how the project will genuinely benefit local people.

“It’s almost like, you know, research is an industry that creates this very individualised approach to the work that’s been done. So, the researcher comes up with the research question, they design the methods, they, you know, conduct the research, and then they write it up, it’s all very individual focused and often driven around somebody’s qualification or a research grant, whether they’re trying to get a PhD, or, you know, that they’ve been successful in a research grant. And I think what we’ve tried to do is shake that up a bit, and try and get researchers to understand that they don’t have all of the answers, that they’re not always the experts, and that they should be looking to value add, rather than come in and save us. And so, I think there’s been some really positive experiences, but there’s also been some really frustrating experiences where people, you know, can’t seem to break the mould of the traditional researcher. And that takes a lot of energy and a lot of effort. And yeah, can be quite frustrating.”

(Participant 6)

Conversely, three interviewees spoke about positive engagement experiences where researchers offered their services without saying what it had to be for, and not necessarily as a research project. One interviewee spoke of an Aboriginal researcher with a long-standing relationship, leading an Aboriginal research team, who engaged with the organisation early with only a general research idea. Another interviewee spoke of the need for ongoing education in this space: “There’s still a lot education to be done with external stakeholders around research and about co-designing, and I mean co-designing, physically sitting down together and deciding what the design is, as opposed to the design’s already done … That’s still a long way to go” (Participant 10).

Two interviewees spoke about guiding researchers through regional processes, including ethical review beyond that provided by universities. Another interviewee spoke about the inadequacy of national ethics approval processes in the local context. Further to this, some interviewees said there had been some improvements over the past 15 years with the introduction of the KAHPF Research Subcommittee. Several interviewees recounted ethics-related concerns, including feeling pressure from their funding bodies to participate in some projects; incomplete or inaccurate paperwork in ethics applications, which added to the administrative burden; lack of clarity in consent forms; and unclear project start and finish dates. One interviewee spoke about a “new wave” of a “long” research project that had substantially different methods, and the involved health service had the responsibility for ensuring that the interviewees gave properly informed consent and were aware that it was not part of routine care. Other interviewees commented that the same study involved a burden on the clinic with minimal assistance from the research team for its day-to-day running. While most researchers in the interviewees’ stories were non-Indigenous, two interviewees also spoke about being disappointed and frustrated by the behaviours of some Aboriginal academic researchers. 

An Aboriginal participant noted that fulfilling ethical responsibilities should not be “such a tick a box process” and should include “following up with [community research partners] and, you know, making sure that people feeling heard and valued … And yeah, how we hold these participants and how they’re acknowledged as well” (Participant 1). The same interviewee spoke about a local Aboriginal person being employed as a “cultural navigator” without being genuinely utilised, which the interviewee described as the researchers “tick[ing] their Black box” (Participant 1). This interviewee also recounted examples of attending a workshop and not realising it was a research project until they arrived there and then felt compelled to participate. This interviewee also recounted substandard practices in a project by a senior non-Indigenous researcher with decades of experience “in the Aboriginal space”. Rather than taking responsibility, the researcher described themselves as “just a stupid white fella” and that they needed Aboriginal people to “pull me up about these things” (Participant 1). Another interviewee mentioned the need for clear guidelines and expectations regarding cultural security and ethical standards to ensure cultural safety is maintained at all times. Two Aboriginal interviewees discussed the need for people to know what their rights are around ethical conduct and mentioned KAHRA being a great potential support in this area for health services and community members, including to help “change our people’s perception of what research is and have more involvement” (Participant 11).

### 3.2. Translation of Findings and Impact of Research

Many interviewees stated that researchers either did not present findings, including reporting and feedback on progress, or presented findings without noting outcomes or practical implications.

“You really need to be thinking about the way that you’re communicating about this work. Because it’s not resonating with us on the ground, the amount of times we’d come to these presentations and meetings, and they would leave and we’d all kind of go, so what are they doing? Like, they just have such a big picture view of it or something that I just can’t seem to distil it down into these just really practical chunks of information, or I don’t know where the disconnect is, because it’s not like they don’t know what they’re talking about. And it’s not like it actually isn’t useful … But then if they’ve got more community involved, then that will help. They will be able to provide that feedback. And you know, how to communicate it in ways that people could connect with.”

(Participant 1)

Reported lack of feedback to health services and communities was consistent with more than 10% of all projects having an “indeterminate” status due to researchers ceasing communication with the KAHPF Research Subcommittee (Table 1). One participant noted that feedback to the community was an ethical responsibility. As shown in Table 2, a number of projects have been of a lengthy duration. One participant mentioned the provision of preliminary findings before the end of a project was valued, in contrast to other research where “You see it sit on the shelf … no outcomes coming out of it and not involving the people being researched and telling them what the findings are about” (Participant 11). This participant also stated: “I want more Aboriginal people be leaders in this research, being chief investigators, getting more involved with getting the feedback, and then what are the pathways to follow up on the feedback? … But there needs to be some recommendation, the findings are different [to current understanding] … this is what we recommend you as a community should be doing” (Participant 11).

One interviewee commented on research with practical components and outcomes being the most useful kind of research: “Having the two in combination is better than just someone getting information and going” (Participant 15). As shown in Table 2, most completed and current projects appeared to have practical (action-oriented) purposes, such as improving health services and health care. However, 50 out of 172 current or completed projects (29%) were descriptive only (no practical outcomes as part of the project). Two other interviewees explained how they design their projects to have “immediate and direct benefit” (Participants 7 and 8) for their community, enabling them to assist people throughout the project, not just when the findings are reported. They also commented on how they “weren’t going to do research for the sake of research” and having local Aboriginal organisations leading projects on the ground ensures they are responsive to the needs of the community. Along with other interviewees, they also mentioned the benefits of ongoing follow-up and assistance being continuously provided to the community. “We’re actually building the data and the story so people can have some really good insight into what’s needed … it’s to determine our future, really, and for our community” (Participant 7).

As shown in Table 1, more than 100 projects had been completed during this 15-year time period. The most tangible outcomes reported by interviewees were improved equipment and/or processes, including clinical protocols that came from a total of four research projects mentioned in their interviews. Another participant noted that “there’s a list of outputs beyond papers” (Participant 8), including engaging with the government and presenting to parliamentary inquiries, and others perceived that the purpose of various projects was “to use it to influence policy and funding” and “build the evidence to show the impact that it’s having on improving wellbeing outcomes and use that to leverage for additional resources and on investment” (Participant 6). 

Four interviewees noted that research was good for learning but that money was required to be able to act on findings. Expanding on this sentiment, one participant mentioned that there were not enough resources built into projects to translate and implement the findings from the project on the ground. Similarly, another participant commented on how funding, governments, and academia do not currently allow for adequate co-design, “good translation going back to community” (Participant 6), and implementation of project findings. In addition, this participant commented on the unnecessary duplication of research projects over time due to a lack of adequate translation and implementation of findings and the varied benefits that can come from research that is done properly.

“There’s the benefit of empowering communities to be involved in research. … There’s the ability to engage community in doing research and upskilling community in research and also providing an income for people to do research. There’s the benefits of improved practice and improved clinical outcomes … the benefits of building the efficiency and effectiveness of health services and our ability to improve health outcomes … it’s largely dependent on the quality of the research design as to whether or not you maximise those benefits along the way.”

(Participant 6)

### 3.3. Local Involvement and Control

Alongside embedding research translation from the beginning with greater transparency, a number of interviewees noted the importance of local people being engaged throughout research projects, with consideration of existing demands on health service staff and Aboriginal leaders. The locations of projects submitted to the KAHPF Research Subcommittee included approximately 20 communities across the Kimberley and the six towns. Most projects (62%) involved multiple locations within the region. Interviewees emphasised the importance of local Aboriginal community navigators and the potential benefits of a more coordinated, long-term approach to building the local Aboriginal research workforce. 

Several interviewees spoke about the expectation placed on them to assist with research projects as part of their actual job without any additional resourcing, and one noted that research projects were sometimes considered by researchers to be the priority over the organisation’s funded core business. They noted that even with resourcing, suitable staff may not be available to assist with research projects. One participant expressed their hopes for research in the Kimberley to build knowledge “that is owned, controlled, and governed by Kimberley Aboriginal people” (Participant 12), with appropriate research approval processes, local workforce, and regional research principles. Other interviewees commented on the need for communities to be setting their own research priorities. Most projects until the end of 2020 (60%) were not initiated from within the Kimberley. At least 37% had no Kimberley-based investigators listed (Table 1). During this time period, the Aboriginal status of investigators was not consistently collected (missing data for 63% of projects, Aboriginal investigator/s recorded for 30% of projects). An increase in project applications between the two 7-year periods, 2007–2013 and 2014–2020, was largely attributable to projects initiated from outside the Kimberley; there was an increase of 26 project applications in the later period, with an increase of five projects initiated in the Kimberley. Some interviewees expressed confidence in saying no to proposed projects that will not fit or be of benefit to their community, noting that what may work in one Kimberley community may not work in another community.

There were multiple examples of research-related capability within the region, including within “role model” (Participant 2) health services; many Aboriginal people with formal research training and extensive experience, knowledge, and skills; collaborations between local organisations; and local Aboriginal people presenting research findings at global forums. 

Two interviewees spoke about successful partnerships between Aboriginal-led organisations and researchers employed by universities in other states. The Aboriginal organisations and key community leaders maintained ownership and leadership of partnership activities. Aboriginal leadership included ensuring research priorities arose from within the community; recognising and addressing the need for foundational community education prior to the research project commencing; insisting on building relationships first; genuinely respecting and connecting with participants; directing the use of research funds for local Aboriginal community navigators and other roles to be involved in all research activities; requiring that researchers lived in the community to have a true understanding of the context; key Aboriginal leaders having the power to say no; and asserting that they “wanted to own the research afterwards” (Participant 7). These interviewees also spoke about the effectiveness and increased engagement of a program that is led by Aboriginal people: “It’s the difference, and you can feel it” (Participant 8).

Two other interviewees spoke about their organisation’s involvement being crucial for a project, alongside the community, although they were not asked to be involved at the beginning.

“They [the researchers] found to make it work we had to be part of it … we made it easier for them.” These interviewees then continued to explain how this involvement sometimes plays out: “They get their foot in the door, get the contacts and then they’ll, once they get to know their way around a bit you sort of feel like they sort of, ‘well, thanks very much, but we can do it on our own’ … They gotta respect people, people’s time, and even interviewing community people … there’s a lot of things that come out of it, they sort of take for granted because it’s a community or whatever.”

(Participant 15)

Other interviewees spoke about their involvement in a research project enabling better qualitative data:

“I think it’s because we sat in with the interviews with the actual clients, gave them more, sort of, made them feel that they were actually heard, rather than sitting with just a doctor or a researcher, had no idea what they were talking about. … So, I think that was the most important thing is actually sitting in with the clients, patients. Making sure they understood what the researchers were trying to think. And in saying that, the interviews were only done by the health workers, not by the researchers or the doctors … and there was no wrong or right about it [for participant responses], it was being heard by the Aboriginal staff.”

(Participant 2)

One participant discussed how there has been a lot of Aboriginal-led research with good outcomes, but they do not sell themselves well and are not truly recognised for the essential knowledge they contribute.

“It tends to be that the researcher presents it as opposed to us presenting it … And that needs to change … because the researcher can’t do their work if we’re not involved. … That’s your job as a researcher, but you got the knowledge from this person over here? Yes, that’s the bit they can’t sometimes comprehend. Because you got to live it and breathe it every day. And they’re getting their information from yous, … I think it’s just changing the culture of the way research is done. Because if you Google everything that’s done on Aboriginal people, back in the day, there’s no Aboriginal names there. It’s all non-Indigenous people that have done the research, but all our data sovereignty, all [our] information, all [our] lifestyles. That’s the bit we want to change.”

(Participant 10)

Other interviewees spoke of personally experiencing a lack of acknowledgement for their contributions to research projects.

“I find that really concerning, I think, all the work that I’ve been involved in and not been acknowledged for in any way, shape, or form. … I’ve done significant amounts of work on [projects] and not been offered any form of authorship at all, … my track record right now, it could be [extensive], like the amount of projects that I’ve been involved in.”

(Participant 1)

Conversely, one participant spoke about the value of being acknowledged through authorship in an academic journal article.

“I think now that it’s sort of out there, the research has been done. And the article that’s out there, so we’re sort of, we took ownership of it, which gives us, sort of make us feel proud of that, you know, this is ours, you know, we take ownership of it now. This is going to be with us forever, and handed down to our next generation. And they know this is a, Nana, Lulu [grandfather] and Uncle, Aunty, they, they did this for us. This is where we are today. So, the acknowledgement … of what we done is sort of go down in history, I’d say in history through future health workers and practitioners.”

(Participant 2)

Many interviewees mentioned inadequate funding of projects as a barrier to effective research. “If you don’t have adequate funding, then you either don’t do anything, or you are a burden” (Participant 9). Interviewees explained the importance of local knowledge for adequately resourcing projects, noting high travel costs, with additional resources required due to remoteness and local knowledge required for feasible travel plans: “They’ve just got no idea” (Participant 15).

“[It’s] different to working in a lab or in a city environment. And that all those factors have to be in there and generally cost money … I don’t own a petrol station and I don’t own fleet of cars, I got to pay for them. So, when you use them they cost money, so that’s a different issue … you might have a few synergies with wages, or while I’m there, can I go in, you know, clock on somewhere, that’s fine. But when you first specifically kind of drive there for that project, then it becomes a cost. I think that’s that common sense sort of stuff. … [For another project] we’re catching an aeroplane to go and do it, I don’t own an aeroplane [laughs]. We are not pilots, it costs money. The thing is, the part of we feel obligated to take part in, looking after our community.”

(Participant 14)

One participant expressed how research resourcing could improve practical service delivery, including resourcing practical community-based roles within a research project. Others noted that research projects that bring staff or other resources into communities do not pose as much burden but involve difficulties with sustained systems change. One interviewee expressed that we do not necessarily need more research to improve outcomes, we need “longevity and programs to make those things better” (Participant 15). Other interviewees discussed the value of research evidence in advocating for funding essential services and programs on the ground, that the government was not providing, and to better align services to meet the needs of communities.

“Long term funding [is needed for organisations on the ground], because the other thing is, we’re not going anywhere. We were born from the people … that’s our, Fitzroy Valley is our home. So, we got our families there, we’re connected to the place … for an Aboriginal organisation, we’re not going anywhere, we have a stable workforce, we’re not like government where they 3 years gone, then you’re doing new one. So, we have to be, we’ve got to be seen for the good work that we do do. And that we’ve got to be resourced properly for it.”

(Participant 7)

One participant noted that although resources and support are needed, much can and should be started from within health services and local communities: “It’s not going to be done by someone from Perth or Melbourne” (Participant 6). 

The topics of all projects submitted to the KAHPF Research Subcommittee are shown in Table 3. When interviewees were asked what they would like to see researched, most ideas involved social and emotional well-being and mental health, environmental health, and the social determinants of health. Most importantly, however, interviewees noted these projects needed to come from within, and be led by, the community. Details of research ideas have been outlined in a report for Aboriginal Community Controlled Organisations, Aboriginal Community Controlled Health Services and other health service stakeholders. 

## 4. Discussion

The findings of this project speak for themselves and are a powerful portrayal of inappropriate research behaviours that have continued over many years. This is directly related to the insufficient positive impact of the large amount of research conducted in the region. It is clear that local knowledge and expertise in research are already established in the region and should be recognised and respected by researchers. A notable example of Aboriginal-led research excellence was evident in the Fitzroy Valley, providing a model for how proper co-design and research collaboration under Aboriginal control must be done.

The themes highlighted in this study are closely aligned with documented knowledge of the past research landscape in Australia [6,9,10,11,13,26,27,28]. Although we did not ask for negative stories from the interviewees, the fact that each expressed similar sentiments and hopes for research into the future, even with their vastly different backgrounds, was notable and provided clear direction. The concerns of interviewees and quantitative findings in this project are also consistent with numerous other documents that discuss colonising ways of research, lack of acknowledgement of Aboriginal people, research being conducted ‘on’ Aboriginal people, and lack of action research projects [5,11,14,29]. Humphrey [10] discussed the lack of action-oriented projects and institutional arrangements, including funding, being an issue; these are still issues in the current study more than two decades later [30]. The themes in this current study are also similar to those from the study that focused on KAHPF Research Subcommittee stakeholders until 2013 [13], and externally-driven research demand has increased since then. This demonstrates that not enough has changed and more needs to be done. There were many points of difference in the current project, including Aboriginal oversight and strength in capturing the voices of local people impacted by research practices in the region. 

Some positive partnerships between local organisations and external researchers were evident in this study. It is clear moving forward that research agreements between equal partners need to be defined and adhered to [31]. Alongside this, consideration must be given to comments from interviewees that even Aboriginal people conduct Aboriginal health research inappropriately at times, indicating issues with academia and its expectations [32]. An associated issue is that government agencies and funding bodies grant money to well-written, compelling research applications that are not necessarily wanted by or beneficial for the community that the research is intended for. This study found that government-funded projects conducted by researchers external to the Kimberley have previously neglected to share their findings within the region. This is expected to improve for the future as part of Closing the Gap Priority Reform 4—Shared Access to Data and Information at a Regional Level [33].

This study had several limitations. Due to the incomplete records used for the quantitative component of this project, limited emphasis was placed on this data. Data limitations also impacted our ability to investigate the outcomes of research. We had originally intended to use KAHPF Research Subcommittee annual and final reports as part of analysing project outcomes, but the availability and quality of this data were insufficient. The KAHPF Research Subcommittee application form had also changed over the years resulting in some data gaps. Aboriginal investigators were not captured specifically on application forms until KAHRA took over the secretariat function in 2021. Originally, further progression in the development of research priorities from local Aboriginal-controlled organisations in the Kimberley was expected so they could be compared with our findings, but this was delayed due to COVID-19. Research ideas suggested by interviewees will be used to assist in the continued development of these local priorities. Some relevant health research projects were not submitted to the KAHPF Research Subcommittee and were not captured in this project, as resourcing constraints precluded additional investigations beyond Subcommittee records. In 2021, the KAHPF Research Subcommittee and WAAHEC clarified that the holistic definition of health used by the National Aboriginal Community Controlled Health Organisation (NACCHO) is relevant for Aboriginal health projects [34]. 

A major strength of this project was Aboriginal investigator involvement and oversight in every aspect of this project [35]. More time and resources need to be embedded in projects for this to be the minimum standard practice in all research. The novice researchers in the research team were horrified to see how easy it would be to interpret data differently, with a different lens to that of what the interviewee intended if the correct people were not present at all stages of the project. It is not possible for a non-Indigenous person to analyse the data accurately without an Aboriginal person connected to the participants or their context [32,36]. Another strength was rich qualitative data, including from Aboriginal leaders. The relational accountability of the research team created comfortable and honest discussions in a trusted space with the participants.

This study’s findings are an important contribution to the creation of a Kimberley Aboriginal health research model to be developed collaboratively with Aboriginal Community Controlled Health Services in the Kimberley. This model will utilise information from many different sources, including building on expectations and approaches that already exist in the Kimberley. It is envisaged that this model will form the basis for the assessment of potential projects in the region, noting that regional support is a requirement prior to receiving WAAHEC ethics approval. Recommendations for this model are included in Table 4:

Other recommendations from the findings include small, community-led projects, completed and implemented, with practical outcomes, in a reasonable timeframe. Almost every interviewee mentioned environmental health as a major health concern in the Kimberley and one that more research needs to be conducted on. While 24 projects were conducted regarding various infections, only seven projects were conducted on environmental health—the majority of the infections studied are ones that are caused by environmental conditions. This highlights a need for more focus on prevention.

## 5. Conclusions

The Kimberley region has finite resources for research: time, personnel, clinic availability, and potential participants. From our findings relating to the past 15 years, it is clear that moving forward, health research conducted in the Kimberley needs meaningful participation, leadership, and ownership by local Aboriginal people. There must be a move from researchers requesting health services and communities to be involved in researcher-driven projects to researchers offering up their services for projects developed within the community according to the community’s needs. Knowledge translation needs to be improved as a basic first step to increase impact and practical benefits for communities across all research endeavours. If every project conducted in the Kimberley was carried out this way, there would be a vastly different health landscape. Advocacy is needed for government and other funding bodies to include adequate funding for engagement, co-creation, knowledge translation, all necessary research activities, and data. A coherent research strategy is needed, produced together with local organisations, with targeted impacts and benefits for Aboriginal people. Fundamentally, Aboriginal health research is meant to improve Aboriginal people’s health and well-being, and this needs to be the priority for anyone working in this space.

## Figures and Tables

**Table 1:** Characteristics of projects submitted to the KAHPF Research Subcommittee until the end of 2020.

Characteristic of Submitted Project	*N*	%
Projects submitted each year		
2007	6	2.6
2008	12	5.2
2009	12	5.2
2010	15	6.5
2011	23	10.0
2012	18	7.8
2013	16	7.0
2014	18	7.8
2015	21	9.1
2016	16	7.0
2017	28	12.2
2018	16	7.0
2019	12	5.2
2020	17	7.4
Total	230	100.0
Project status at end of 2020		
Completed	106	46.1
Current	66	28.7
Indeterminate	27	11.7
Started but not completed	3	1.3
Not started—not assessed	2	0.9
Not started—not supported	2	0.9
Not started—supported	23	10.0
Not started—unknown if supported	1	0.4
Initiated in Kimberley		
Yes	87	37.8
No	139	60.4
Missing	4	1.7
Kimberley-based investigator/s		
Yes	141	61.3
No	86	37.4
Missing	3	1.2
Funding		
Australian Government department	33	14.4
Australian Research Council	4	1.7
NHMRC	41	17.8
Other Australian Government agency	3	1.3
WA Government department	12	5.2
WA Government agency	12	5.2
Lowitja Institute	2	0.9
Other not-for-profit organisation	15	6.5
University/research institute	12	5.2
Other ^1^	10	4.3
No funding recorded (missing data)	86	37.4

NHMRC = National Health and Medical Research Council. KAHPF = Kimberley Aboriginal Health Planning Forum. ^1^ Includes philanthropic foundation/donor, company, or unspecified funder. Percentages may not add up to 100 due to rounding.

**Table 2:** Characteristics of completed and current projects with KAHPF Research Subcommittee approval, December 2020.

Characteristic of Current or Completed Project	*n*	%
Approximate duration (calendar years)		
Completed projects		
<1 year	8	7.6
1 year	26	24.5
2 years	29	27.4
3 years	17	16.0
4 years	8	7.6
5 years	7	6.6
6 years	2	1.9
Unknown duration (missing data)	9	8.5
Total	106	100.0
Current projects (at end of 2020)		
0–3 years	47	71.2
4–6 years	13	19.7
7–9 years	3	4.6
>=10 years	3	4.6
Total	66	100.0
Purpose ^1^		
Descriptive	80	32.1
Detection of health condition	21	8.4
Management of health condition	22	8.8
Prevention of health condition/s	21	8.4
Service improvement	70	28.1
Evaluation of program or intervention	35	14.1
Total projects purposes (172 projects)	249	100.0

KAHPF = Kimberley Aboriginal Health Planning Forum. ^1^ Some projects are included in more than one category. Of the 172 current/completed projects, 50 had a wholly descriptive purpose (29.1%). Percentages may not add up to 100 due to rounding.

**Table 3:** Health topics of projects submitted to the KAHPF Research Subcommittee until end 2020.

Topic	*n*	%
General health	11	3.8
Alcohol	6	2.1
Drugs	4	1.4
Smoking	5	1.7
Healthy eating	10	3.4
Physical activity	3	1.0
Chronic disease ^1^	39	13.4
Cancer	3	1.0
Ear health	8	2.7
Eye health	5	1.7
Oral health	2	0.7
Infection ^2^	24	8.2
Environmental health	7	2.4
Social determinants	7	2.4
Cultural determinants	10	3.4
Medication	3	1.0
Brain injury	3	1.0
Stroke	3	1.0
Disability	2	0.7
Mental health (including depression, anxiety, trauma)	24	8.2
SEWB (including healing, resilience, and wellbeing)	34	11.7
Sexual health	19	6.5
Pregnancy	18	6.2
Postpartum	7	2.4
Parenting	3	1.0
Child neurodevelopment	8	2.7
Workforce	14	4.8
Other	9	3.1
Total project topics (230 projects) ^3^	291	100.0

SEWB = Social and Emotional Wellbeing. ^1^ Includes diabetes (*n* = 12), rheumatic heart disease (*n* = 11), other heart conditions (*n* = 4), kidney disease (*n* = 3), lung conditions (*n* = 6), other chronic diseases (*n* = 3). ^2^ Includes acute rheumatic fever (*n* = 3), skin infections (*n* = 5), and other infections (*n* = 16). ^3^ Some projects are included in more than one category.

**Table 4:** Recommendations for a Kimberley Aboriginal health research model.

Recommendations from This Study	Practical Examples and Accompanying Literature
Research that is led, developed, driven, and owned by the community	Research projects must come from within the community, not from the researchers. Partnerships can be requested from local organisations if particular expertise is required [37].
Local Aboriginal leadership	Local Aboriginal Community Controlled Organisations are well placed to lead health research with their communities [9].
Alignment with Kimberley research priorities	Further work across the region is required to refine and coordinate Kimberley research priorities and ensure that research conducted in the Kimberley is wanted by Kimberley people [6].
A requirement for the crucial contributions of Aboriginal people in research projects to be fully recognised.	It has been well documented that Aboriginal people must be acknowledged for their significant contribution to research [7].We will take this a step further by supporting the acknowledgement of Aboriginal people in the most prominent authorship positions.
National projects must have local Aboriginal representation on the ground	Employing community navigators/research officers/investigators to consult with the community and conduct the research in the community [28].
A comprehensive knowledge translation plan to be embedded in all projects	Some examples from the Lowitja Institute include: “thinking beyond the project, and building knowledge exchange into the research plan; working with the people and organisations that use research findings; looking beyond the academic world when disseminating research findings; good communication, real relationships, and practical approaches” [37].

## Data Availability

The data from this study is unavailable due to privacy or ethical restrictions.

## Appendix A

**Table A1:** Interview guide.

Domain	Questions and Prompts
Context	- Can you detail what your involvement has been in research in the Kimberley? - Can you detail any previous research experience you may have?
Process	- How did the researcher get in contact with you? Was this early in the research process? - What has this experience been like for you and your organisation? - Do you feel like your organisation’s values and ideas were respected and adhered to? - How was the purpose and benefit of the research explained to you? - Can you tell me about communication from the researchers, during and after the project?
Impact	- What benefits or impact have you seen come from the research conducted within your organization or your community? - New programs? Practice?
Future	- What changes would you like to see in research in the Kimberley? Can you tell me about your ideal research process? - Can you detail any gaps in research in your area?

**Table A2:** Additional quotes from interviewees.

**Theme**	**Illustrative Quote**
Questionable ‘research world’ behaviours	“What’s common process in the Kimberley is the researcher sort of comes up with a research idea, and then comes to the Kimberley seeking support to do it, and they come to the Aboriginal medical services, usually to say, I’ve got this great idea, will you write a letter of support, because it’s a requirement of ethics … So that’s normally the traditional approach. I think in more recent years, we’ve seen people come and say, you know, this is my area of expertise, is there any opportunity for you guys to be involved much earlier and design the research … sometimes we felt quite cornered, that we had to, you know, agree to research because it had already been funded. But we could also see the benefit of it. And then there were other instances where we were able to stop research, if we didn’t think it was meaningful, or, you know, was going to take more effort or more time than the benefits back to health services or the community … The establishment of KAHRA [Kimberley Aboriginal Health Research Alliance] hasn’t been a research project in itself, it’s been a recognition that we need to be doing research differently.” (Participant 6)
Translation of findings and impact of research	“Definitely like the hugest, hugest gap when I was, so many, but the one that really stood out to me was that the presenting findings back to us it just never happened, it never happened. Like, it was just shocking.” (Participant 1)
Translation of findings and impact of research	“You can’t ask us to be involved and then you don’t come and see us and we’ve got all our staff involved in it, but you don’t see it till the very end.” (Participant 10)
Local involvement and control	“I think, what’s the important characteristics of any research is that the design of it is co-designed with health services and communities so that it’s the benefits that there’s real clarity around the purpose of the research and the opportunity to maximise the benefits from the research, come out of that kind of early engagement process … [in one project] by engaging early, you know, the sector was able to say, Hang on a second, don’t just waste your time, if you’re going to have these people in these communities, what are we going to do to give back to the community? What kind of education can we do? How can we try and address the systemic issues here.” (Participant 6)
Local involvement and control	Participant 7: So the lay of the land basically, they needed to know that I wanted them to really get to understand that … that they had to be there— Participant 8: Well, living on, living on communities, when you can go into a community … you can’t have that unless you have Aboriginal organisation or these community, Indigenous community navigators … and the more I think about it, but that’s the only way that research can, can happen.
Local involvement and control	“I think AMSs [Aboriginal Medical Services] back then and we’re talking, you know, more than 15 years ago, probably talking 20 years ago, research was driven by our stakeholders as opposed to us driving the research. I think if you compare it to now, we are in a much stronger position as Aboriginal people to be able to say, ‘No, it doesn’t have a value to us, it’s not going to have an outcome to our people’. And we’re in a position now where there is much more knowledge across the Kimberley, and across our Aboriginal Medical Services, including our ACCOs [Aboriginal Community Controlled Organisations] around driving and putting us first, because, you know, I’ve got to be honest, we’re the most research people in Australia, there is so much information in Australia about us that we were not involved in. And I think we’re in a position now where we can drive better outcomes … the other important bit is that we want more Aboriginal people involved as researchers, we want more Aboriginal people involved in on the advisory councils. We want more Aboriginal people involved in as chief investigators, and they don’t have to be clinical people. We’ve got so many talented Aboriginal people in the Kimberley that you don’t have to be a clinician to do research. So, it’s about giving people the opportunity to be able to take that space.” (Participant 10)
Local involvement and control	“Just trying to explain to them that you just need to build those relationships and meet with people, again, people [on project teams] that are not new to the space, it just seems like I’m banging my head against a brick wall sometimes. So, can you just have meetings to connect with people so that they actually know who you are. And also so … that you as a researcher are held accountable” (Participant 1)
Local involvement and control	Participant 8: We’ve been approached outside of this work by other, other researchers who don’t know about community led research … We listen to them. Yeah. We listen to them. … People are now coming to us and say, ‘Oh, we want to partner with you’. But it’s more about us giving them the kudos, I suppose, it doesn’t— Interviewer: The project is already developed? Participant 8: Yes. Yeah. So, we listen to them. And then we say, ‘Thanks, but no, thanks.
